# Association between Hepatic Steatosis and Obstructive Sleep Apnea in Children and Adolescents with Obesity

**DOI:** 10.3390/children8110984

**Published:** 2021-11-01

**Authors:** Marco Carotenuto, Anna Di Sessa, Maria Esposito, Anna Grandone, Pierluigi Marzuillo, Ilaria Bitetti, Giuseppina Rosaria Umano, Francesco Precenzano, Emanuele Miraglia del Giudice, Nicola Santoro

**Affiliations:** 1 Clinic of Child and Adolescent Neuropsychiatry, Department of Mental Health, Physical and Preventive Medicine, University of Campania “Luigi Vanvitelli”, 80100 Naples, Italy; marco.carotenuto@unicampania.it (M.C.); maria.esposito3@unicampania.it (M.E.); ilaria.bitetti@gmail.com (I.B.); francesco.precenzano@unicampania.it (F.P.); 2 Department of Woman, Child and General and Specialized Surgery, University of Campania “Luigi Vanvitelli”, 80138 Naples, Italy; anna.grandone@unicampania.it (A.G.); pierluigi.marzuillo@gmail.com (P.M.); giusi.umano@gmail.com (G.R.U.); emanuele.miraglia@unicampania.it (E.M.d.G.); 3 Department of Pediatrics, Yale University, New Haven, CT 06510, USA; nicola.santoro@yale.edu; 4 Department of Medicine and Health Sciences, “V.Tiberio” University of Molise, 86100 Campobasso, Italy

**Keywords:** obstructive sleep apnea, fatty liver, apnea hypopnea index (AHI), oxygen desaturation index (ODI), insulin resistance (IR), children

## Abstract

Background: Owing to the increasing rate of pediatric obesity, its complications such as non-alcoholic fatty liver disease (NAFLD) and obstructive sleep apnea (OSA) have become prevalent already in childhood. We aimed to assess the relationship between these two diseases in a cohort of children with obesity. Methods: We enrolled 153 children with obesity (mean age 10.5 ± 2.66, mean BMI 30.9 ± 5.1) showing OSA. Subjects underwent a laboratory evaluation, a cardio-respiratory polysomnography (PSG), and a liver ultrasound. Results: All subjects had a clinical diagnosis of OSA based on the AHI > 1/h (mean AHI 8.0 ± 5.9; range 2.21–19.0). Of these, 69 showed hepatic steatosis (62.3% as mild, 20.3% as moderate, and 17.4% as severe degree). A strong association between ALT and apnea/hypopnea index (AHI) was observed (*p* = 0.0003). This association was not confirmed after adjusting for hepatic steatosis (*p* = 0.53). By subdividing our population according to the presence/absence of steatosis, this association was found only in the steatosis group (*p* = 0.009). As the severity of steatosis increased, the significance of its association with AHI compared to the absence of steatosis became progressively stronger (all *p* < 0.0001). Conclusions: Hepatic steatosis seems to drive the association between OSA and ALT levels, suggesting a potential pathogenic role of OSA in NAFLD.

## 1. Introduction

During the past decades, the growing prevalence of pediatric obesity has been accompanied by a parallel increased prevalence in childhood of its cardiometabolic consequences such as non-alcoholic fatty liver disease (NAFLD), type 2 diabetes (T2D), metabolic syndrome (MetS), cardiovascular disease (CVD), obstructive sleep apnea (OSA), and insulin resistance (IR) [1,2,3]. In particular, NAFLD represents a complex progressive liver condition ranging from simple hepatic steatosis to cirrhosis and liver failure [4]. Of note, the diabetogenic potential of NAFLD has been clearly demonstrated in youths with obesity [5,6]. In fact, NAFLD has been shown to be associated with IR independently of the degree of obesity, and its prevalence has been found to be increased with the increase in both MetS and T2D [5,6]. Noteworthy, the close association of fatty liver with metabolic derangements has been recently emphasized in the new nomenclature of NAFLD as metabolic (dysfunction) associated fatty liver disease (MAFLD) [7,8]. However, some controversy on the effectiveness of this new definition in children with obesity has been reported [9].

Recently, published data have also supported the association of NAFLD with obstructive sleep apnea (OSA) [10,11], a condition affecting up to 27% of obese children [12,13]. In particular, OSA has been linked both to serum alanine transferase (ALT) levels [14,15] and to the degree of steatohepatitis [16]. Furthermore, studies on animal models have reported that chronic intermittent hypoxia, considered as the main OSA characteristic, leads to liver damage likely through the stimulation of oxygen-derived compounds production, which in turn is responsible for the peroxidation of lipids and the activation of pro-inflammatory pathways [17,18,19]. 

Although there is evidence regarding the association between OSA and NAFLD and the role of fatty liver in metabolic derangements development, data on the relationship between NAFLD and OSA in youth with obesity are quite sparse [11,13]. Therefore, we sought to examine this potential relationship in a cohort of children and adolescents with obesity. We also attempted to investigate if the putative link between OSA and ALT might be influenced by the presence of hepatic steatosis. 

## 2. Materials and Methods

A total of 153 children and adolescents with obesity (89 males, mean age 10.5 ± 2.66, mean BMI 30.9 ± 5.1) diagnosed with OSA were enrolled. The main anthropometric measurements (including height, weight, and waist circumference) were assessed as previously reported [20]. The body mass index (BMI) was evaluated, and standard deviation scores (BMI-SDS) were calculated based upon national standards [21]. Subjects underwent a cardio-respiratory polysomnography (PSG). A comprehensive laboratory evaluation (including lipid, transaminase, glucose, and insulin levels) and a liver ultrasound were performed as described elsewhere [20]. In case of persistent (>6 months) increased ALT levels, other liver diseases such as autoimmune hepatitis, Wilson disease, alpha-1-antitrypsin deficiency, hepatitis B and C, and iron overload were excluded.

### 2.1. Cardio-Respiratory Polysomnography (PSG)

All evaluations were performed overnight, starting at the subjects’ usual bedtime until spontaneous morning awakening. The overnight cardio-respiratory polysomnography was performed by Embletta (Embletta X10; Embletta PDS, MedCare Flaga, Reykjavik, Iceland) as previously described [22,23]. All recorded data were analyzed through a device-specific software (Somnologica for Embletta 3.3; Embla, Broomfield, CO, USA) and visually scored by one of the investigators (MC) on the basis of pediatric criteria [24]. 

In particular, obstructive apnea was identified as an airflow cease for at least two breaths associated with paradoxical ribcage and abdominal movements. The hypopnea index was defined as a nasal flow curve signal reduction >50% associated with oxygen desaturation or arousal. Central apnea was defined as airflow absence both at nose and mouth with no inspiratory effort within the duration of the event for 20 s or longer, or two missed breaths at an oxygen desaturation ≥3%, an arousal, or an awakening. The apnea-hypopnea index (AHI) was determined as the number of apneas and hypopneas per hour of sleep [24], and the lowest oxygen saturation value and number of desaturation events ranging from 4% to 90% were computed. Moreover, the oxygen desaturation index (ODI), classified on the basis of events per hour, was assessed [23,25]. A value ≤1 per hour for ODI was considered as normal according to the American Academy of Sleep Medicine (AASM) criteria [26]. A reduction ≥50% in oronasal thermistor signal and a concurrent arousal and/or fall of ≥3% in pulse oxygen saturation (SpO_2_) from baseline were considered to classify hypopneas. Unlike to the common AASM cutoff for hypopnea (as 30% reduction in airflow), we decided to use 50% in an effort to potentially improve OSA criteria. A value of apnea index (AI) > 1 and of AHI ≥ 1.5 per hour were considered as abnormal and used for OSA diagnosis [23].

### 2.2. Hepatic Ultrasound

To assess hepatic steatosis, a trained pediatric radiologist (blinded to all data of the study population) performed a liver ultrasound scanning (Acuson S2000, Siemens, München, Germany).

Hepatic steatosis was assessed by evaluating the presence of an ultrasonographic pattern of a bright liver. According to the ultrasound features, the severity of liver steatosis was classified in four groups: absent steatosis (score 0) was identified as regular liver echotexture; mild steatosis (score 1) as fine parenchymal echoes slightly and diffusely increased with regular visualization of diaphragm and portal vein borders; moderate steatosis (score 2) as moderate and diffuse increase hepatic echogenicity with slightly altered visualization of portal vein borders and diaphragm; and severe steatosis (score 3) as diffusely increased liver echogenicity with poor or no visualization of portal vein borders, diaphragm, and posterior portion of the right lobe [26]. 

### 2.3. Biochemical Measurements

After an overnight fast, blood samples were collected from all subjects. Lipid levels (including cholesterol and triglycerides) were assessed by an enzymatic colorimetric method. Insulin and glucose were assessed by IMX (Abbott Diagnostics, Santa Clara, CA, USA) and glucose oxidase, respectively. To evaluate IR, the insulin resistance homeostasis model of assessment (HOMA-IR) was used: fasting insulin (picomoles per liter) × fasting glucose (millimoles per liter)/135, as previously reported [27]. Serum ALT and aspartate transaminase (AST) were determined by using a Hitachi Analyser (Boerhinger-Mannheim Diagnostics, Indianapolis, IN, USA), as described elsewhere [28,29]. 

### 2.4. Statistics

Continuous variables were tested for normality. In case of non-normal distribution, variables were log-transformed. Before the analyses, Pearson correlations between all the possible covariates were examined to avoid co-linearity (as defined by a correlation coefficient >0.50); none of the examined correlations had a correlation coefficient >0.50. To assess whether there was any relationship between ALT and AHI, a regression analysis was run, and age, gender, BMI-SDS, HOMA-IR, waist, and hepatic steatosis categories (0, 1, 2, 3) were used as covariates. Since the association between ALT and AHI was influenced by hepatic steatosis, a regression analysis between ALT and AHI was run separately in subjects with and without hepatic steatosis. Therefore, multinomial logistic regression was also performed by using hepatic steatosis as a dependent variable, AHI as an explanatory variable, and gender, age, BMI-SDS, HOMA-IR, waist circumference, and ALT levels as covariates to evaluate a possible association between AHI and hepatic steatosis.

## 3. Results

All studied subjects received a clinical OSA diagnosis on the basis of AHI > 1/h (mean AHI 8.0 ± 5.9; range 2.21–19.0). Out of the 153 subjects, 69 showed hepatic steatosis, and of them 43 were classified as stage 1 (mild), 14 as stage 2 (moderate), and 12 as stage 3 (severe). The main characteristics of the cohort are shown in Table 1. 

There was a positive and significant association between ALT levels and AHI independent of age, gender, BMI-SDS, waist, and HOMA-IR (r^2^ = 0.10; *p* = 0.0003). Moreover, when the model was adjusted for hepatic steatosis, the association between AHI and ALT levels was not confirmed (*p* = 0.53). In fact, when the population was divided according to the presence/absence of hepatic steatosis, there was a significant association between AHI and ALT only in subjects with hepatic steatosis independent of age, sex, BMI-SDS, waist, and HOMA-IR (r^2^ = 0.071; *p* = 0.009), but not in those without steatosis (r^2^ = −0.0097, *p* = 0.38). Moreover, we found a significant association between ODI and ALT levels (r^2^= 0.15, *p* = 0.03). However, this association was not significant when adjusted for confounding factors (age, gender, BMI-SDS, waist circumference, and HOMA-IR) (*p* = 0.21). 

Furthermore, we observed an association between AHI and the degree of steatosis independent of age, sex, BMI-SDS, IR, waist circumference, and ALT levels (Table 2). 

As the severity of hepatic steatosis increased, the significance of its association with AHI compared to the absence of steatosis became progressively stronger, with *p*-values ranging from 2.02 × 10^−4^ to 1.20 × 10^−5^ (Table 2).

No significant associations were observed between polysomnographic parameters and HOMA-IR.

## 4. Discussion

In this study, we first showed that the association between ALT levels and AHI seems to be modulated by the presence of ultrasound-detected hepatic steatosis. Moreover, hepatic steatosis, per se, seems to exert a strong association with AHI independent of obesity degree and fat distribution in this cohort of children and adolescents with obesity.

Previous studies in adults have reported an association between OSA and liver injury [10,11,13], as also observed in a large pediatric cohort by Gozal et al. [14]. The influence of intermittent hypoxia on ALT levels and lipid peroxidation was also confirmed in animal studies [14], by suggesting a major role for chronic intermittent hypoxia in oxidative stress. Studies in leptin-deficient mice (ob/ob) have shown that chronic intermittent hypoxia was also responsible for a 30% increase in intra-hepatic lipids by up-regulating genes involved in hepatic lipogenesis [30]. Therefore, the chronic intermittent hypoxia of OSA subjects might favor not only liver injury but also hepatic lipogenesis, in turn closely linked to hepatic steatosis development. This seems to be consistent with our observation reporting an association between hepatic steatosis and OSA independently of the degree and distribution of obesity. In fact, herein we showed that the association between hepatic steatosis and AHI is independent of BMI-SDS and waist circumference. As observed in animal models, this finding might also suggest in humans a potential, pivotal pathogenic role of chronic intermittent hypoxia experienced by OSA subjects in favoring hepatic fat accumulation, likely by up-regulating lipogenic genes [31]. 

More, our data also seem to have a pathophysiological significance. In fact, the association between OSA and ALT (as constant marker of liver function) [32] in our population has been demonstrated as not independent of the degree of hepatic steatosis. Given that, the deleterious effect of OSA on liver histology depends on the presence of fatty liver, by assuming that OSA might represent a second hit leading fatty liver toward NASH. This is also suggested by studies showing that high-fat-induced obesity is required in order for the chronic intermittent apnea to trigger the inflammatory response leading to NASH [16,30]. 

Noteworthy, the association between fatty liver and AHI in our cohort was also found to be independent of waist circumference. Commonly, waist circumference as trunk fat index has been closely related to liver injury [33]. As the strong relationship between waist circumference (as a robust surrogate marker of visceral fat) and OSA was previously reported [20], it could be hypothesized that the association of waist circumference with OSA in the present study was driven by the presence of hepatic steatosis. However, the latter was not taken into account in the analyses, but we acknowledge that it is difficult to dissect the association between those compartments and OSA without a thorough assessment of the fat distribution by using state-of-the-art imaging techniques.

Our study has some strengths and limitations that should be mentioned. The large number of PSG, the state-of-the-art technique to assess OSA, the ethnical homogeneity of the sample (Italian children with obesity), and the young age of the study participants, which eliminates any confounders due to aging (e.g., alcohol intake), are considered as strengths. On the other hand, we are aware of certain limitations of the study. Firstly, the use of ultrasound instead of magnetic resonance imaging (MRI) and the lack of liver biopsies to assess hepatic steatosis should be acknowledged as limitations. Nevertheless, in recent years Shannon et al. compared ultrasound imaging to liver biopsy, by demonstrating that ultrasound had 80% sensitivity and 86% specificity to detect moderate hepatic steatosis and 100% specificity for severe steatosis. Liver ultrasound patterns seemed also to be significantly correlated with the NAFLD activity score [34]. Moreover, the lack of a control group and the use of waist circumference as visceral fat measurement instead of a more sensitive index (e.g., MRI, controlled attenuation parameter) for liver fat assessment represent further limitations.

In conclusion, we have shown a strong association between OSA and hepatic steatosis in children and adolescents with obesity. In addition, the relationship between OSA and liver damage seems to be driven by the presence of hepatic steatosis, by suggesting a pathogenic role of OSA in pediatric NAFLD development and progression.

## Figures and Tables

**Table 1:** Main features of the cohort based on liver steatosis degree.

	Hepatic Steatosis Degree				*p*-Value
	0	1	2	3	
N	84	43	14	12	
Age (years)	10.25 ± 2.77	11.07 ± 2.42	9.31 ± 1.71	11.91 ± 2.91	0.03
Gender (M/F)	47/37	28/15	8/6	6/6	0.71
BMI-SDS	5.26 ± 1.85	5.88 ± 1.96	6.07 ± 1.97	7.14 ± 2.99	0.01
Waist (cm)	88.59 ± 9.31	94.02 ± 11.07	90.36 ± 13.06	105.08 ± 11.84	<0.0001
	Glucose, Triglycerides and liver function				
Glucose (mg/dL)	83.52 ± 6.73	82.46 ± 7.48	81.93 ± 8.85	83.75 ± 11.75	0.80
Insulin (uU/L)	24.50 ± 13.53	31.08 ± 20.56	16.93 ± 7.52	37.61 ± 15.02	0.001
HOMA-IR	5.08 ± 2.87	6.36 ± 4.22	3.53 ± 1.80	8.01 ± 3.76	0.003
Triglycerides (mg/dL)	85.15 ± 42.06	91.53 ± 28.38	99.43 ± 42.94	99.08 ± 35.56	0.13
ALT (UI/L)	31.00 ± 18.79	37.26 ± 31.78	65.5 ± 55.25	55.58 ± 19.89	<0.0001
AST (UI/L)	26.12 ± 10.79	26.36 ± 10.18	38.5 ± 21.65	27.75 ± 6.00	0.004
	Polysomnography derived measures				
AHI (episodes/h)	6.26 ± 5.35	7.68 ± 2.12	11.3 ± 1.56	17.4 ± 10.5	<0.0001
ODI (episodes/h)	4.44 ± 2.53	4.81 ± 2.54	6.73 ± 3.01	7.35 ± 5.08	0.0004
Mean SpO_2_	96.4 ± 1.67	97.2 ± 1.63	97.2 ± 1.60	96.4 ± 0.71	0.07
Nadir SpO_2_	91.5 ± 2.90	90.9 ± 2.40	92.1 ± 2.35	90.6 ± 2.29	0.35
mdes SpO_2_%	5.54 ± 1.38	6.97 ± 1.41	6.77 ± 1.41	6.16 ± 1.43	<0.0001

AHI: apnea/hypopnea index; ALT: alanine aminotransferase; AST aspartate aminotransferase; BMI-SDS: standard deviation score of Body Mass index; HOMA-IR: Homeostasis Model Assessment of Insulin Resistance; ODI: oxygen desaturation index. SpO_2_: pulse oxygen saturation.

**Table 2:** Results of multinomial logistic regression examining the relationship between AHI and severity of hepatic steatosis. Significant *p*-values after Bonferroni correction are highlighted in bold.

Hepatic Steatosis Severity	Main Effect of	Estimate	Standard Error	*p*-Value
mild	AHI	5.20	1.40	**2.02 × 10^−4^**
	sex	−0.59	0.44	0.179
	age	−0.14	0.15	0.928
	BMI	0.06	0.17	0.728
	Waist	0.05	0.04	0.177
	HOMA-IR	0.459	0.884	0.603
	ALT	−0.68	0.85	0.419
moderate	AHI	14.32	3.54	**5.13 × 10^−5^**
	sex	0.02	0.80	0.975
	age	−0.24	0.35	0.499
	BMI	0.16	0.34	0.641
	Waist	0.02	0.08	0.761
	HOMA-IR	−7.573	2.890	0.009
	ALT	3.68	1.94	0.058
severe	AHI	19.48	4.45	**1.20 × 10^−5^**
	sex	0.27	1.02	0.792
	age	−0.31	0.46	0.499
	BMI	−0.24	0.51	0.634
	Waist	0.19	0.13	0.144
	HOMA-IR	−3.639	3.960	0.35
	ALT	4.30	2.56	0.094

AHI: apnea/hypopnea index; ALT: alanine aminotransferase; AST aspartate aminotransferase. BMI-SDS: standard deviation score of Body Mass index; HOMA-IR: Homeostasis Model Assessment of Insulin Resistance. Bold means statistical significance.

## Data Availability

The data presented in this study are available on request from the corresponding author. The data are not publicly available due to the presence of information that could compromise research participant privacy.
